# Prevalence and risk factors of diarrhea among young children in Kenya’s drylands: A longitudinal study

**DOI:** 10.1371/journal.pgph.0003998

**Published:** 2026-01-08

**Authors:** Bonventure Mwangi, Valerie L. Flax, Faith Thuita, Joshua D. Miller, Chessa Lutter, Dickson Amugsi, Estelle Sidze, Linda Adair, Esther Anono, Hazel Odhiambo, Stephen Ekiru, Gillian Chepkwony, Monica Ng’ang’a, Albert Webale, Elizabeth Kimani-Murage, Calistus Wilunda

**Affiliations:** 1 African Population and Health Research Center, Nairobi, Kenya; 2 RTI International, Research Triangle Park, Durham, North Carolina, United States of America; 3 RTI International, Nairobi, Kenya; 4 Department of Public and Global Health, University of Nairobi, Nairobi, Kenya; 5 Department of Nutrition, University of North Carolina at Chapel Hill, Chapel Hill, North Carolina, United States of America; North Carolina State University, UNITED STATES OF AMERICA

## Abstract

Diarrhea is the third leading cause of malnutrition and mortality among children under five globally. Environmental and socioeconomic conditions in the drylands of sub-Saharan Africa may increase the risk of diarrhea, yet few studies have examined the factors in these settings. We therefore aimed to estimate the prevalence of diarrhea and identify potential risk factors among young children in Kenya drylands. Data are from a longitudinal population-based study conducted in Turkana County, Kenya. Surveys were implemented across six waves (May 2021 to September 2023) among 1211 households with children under 36 months at baseline. Caregivers reported on household conditions and episodes of diarrhea in the prior two weeks. Prevalence trends were examined by survey zone, livelihood zone, and child age and sex. Multivariable logistic regressions with generalized estimating equations were used to access distal, intermediate, proximal, and immediate risk factors and reported adjusted odds ratio (AOR) together with the associated 95% confidence interval (CI). Diarrhea prevalence declined significantly over time, from 32.1% at baseline to 8.7% at end of the study. Factors associated with higher odds of diarrhea included caregiver alcohol consumption [AOR = 1.28, 95% CI: 1.02–1.60], child malnutrition (wasting: AOR = 1.20, 95% CI: 1.04–1.39; stunting: AOR = 1.40 95% CI: 1.19–1.65; underweight: AOR = 1.29, 95% CI: 1.11–1.49), household shocks (biological: AOR = 1.37, 95% CI: 1.20–1.57; climatic: AOR = 1.18, 95% CI: 0.93–1.50; conflict: AOR = 1.60, 95% CI: 1.40–1.83), and moderate (AOR = 1.25, 95% CI: 1.04–1.50) or high-water insecurity (AOR = 1.46, 95% CI: 1.19–1.81) relative to no-to-marginal household water insecurity. Protective factors included greater child age (AOR = 0.97, 95% CI: 0.96–0.98), receipt of vitamin A supplementation (AOR = 0.77, 95% CI: 0.66–0.89), deworming (AOR = 0.88, 95% CI: 0.75–1.02), and caregiver handwashing after toilet use (AOR = 0.83, 95% CI: 0.70–0.98). These findings highlight the multifactorial drivers of childhood diarrhea in drylands and underscore the need for integrated interventions that improve water security, strengthen nutrition, support hygiene practices, and enhance resilience to household shocks.

## Introduction

Diarrhea, the passage of three or more loose or liquid stools per day, is the third leading cause of death among children under five years of age globally [1]. It is estimated that about 23% of deaths in children under five years of age in Eastern Africa are caused by diarrhea [2]. Diarrhea increases the risk of dehydration, appetite loss, malnutrition, electrolyte deficiencies, infectious disease, delayed physical growth, and cognitive impairments [1,3]. Malnutrition and diarrhea are interrelated, with each condition exacerbating the other, creating a cycle that severely compromises the long-term health and development of impacted children [4,5].

Previous studies in sub-Saharan Africa have identified factors associated with a higher risk of child diarrhea. These include young maternal [6,7], child age [6,8,9], low maternal education [6,10,11], maternal unemployment [6,7], poor hand hygiene [12–14], open defecation [15–17], unsafe fecal management [18,19], and households with more members [6,20,21]. Recent research also underscores the influence of behavioral, sociocultural, and socioeconomic factors on diarrhea risk in rural [22] and urban areas [23]. Given this, interventions that increase access to child-friendly sanitation infrastructure, improve hand hygiene behavior, and expand access to reliable water services have been recommended to reduce the burden of child diarrhea [22,24].

Communities in the arid and semi-arid lands (drylands) of sub-Saharan Africa are particularly vulnerable to infectious diseases. This is largely due to limited access to improved water, sanitation, and hygiene services, which increases exposure to pathogens and the risk of diarrhea [3,25]. Additionally, recurrent climatic shocks such as unpredictable or excessive rainfall, prolonged drought, and famine compromise agricultural productivity and strain critical social and physical infrastructure [26–29]. These shocks contribute to income loss, which in turn limits individuals’ abilities to access quality healthcare [30,31]. Populations relying on nomadic or semi-nomadic pastoralist livelihoods are [30,32–35] acutely impacted, often experiencing concurrent food and water insecurity, which increases their risk of malnutrition and illness [32].

While the distinctive conditions of drylands pose heightened risks for diarrhea, limited research has investigated how environmental (e.g., use of unsafe drinking water sources, poor sanitation and hygiene practices and facilities), socioeconomic (e.g., maternal education, household wealth), behavioral (e.g., proper handwashing during critical times, unhygienic food preparation), and nutrition factors (e.g., child feeding practices) intersect to influence diarrhea risk in these regions, particularly in Northern Kenya. To address this knowledge gap, we therefore aimed to [1] assess how the prevalence of diarrhea changed over six survey waves and [2] identify risk factors for diarrhea among children under five years of age in the drylands of Northern Kenya. We hypothesized that greater reports of household and environmental shocks, higher levels of household water insecurity, poor sanitation and hygiene practices, and child malnutrition would be associated with greater child diarrhea.

## Methods

### Study area

This study was conducted as part of the USAID Nawiri program [33], which aimed to examine dynamics influencing acute malnutrition among children in Turkana and Samburu Counties. For this analysis, we restrict to data from Turkana County, which is in the north-western dry zone of Kenya [34]. The county is divided into four survey zones: the Central zone, consisting of Turkana Central and Loima sub-counties; the North zone, including Turkana North and Kibish sub-counties; the South zone, comprising Turkana East and Turkana South sub-counties; and the West zone, which covers Turkana West sub-county.

Turkana County is characterized by low annual rainfall, high year-round temperatures, and a topographically varied landscape that ranges from semi-arid to arid. Traditionally, the Turkana people are nomadic pastoralists, relying on migration to identify pastures that support livestock and their livelihoods. In addition to pastoralism, some communities practice agro-pastoralism, engaging in crop farming through irrigation along the River Turkwel, and fishing on Lake Turkana [35].

### Study design

A multistage sampling strategy was used to ensure that findings were representative at the regional and sub-regional levels. In the first stage, the population was stratified based on administrative zones. Within each stratum, villages were randomly selected. Study staff conducted a household listing in selected villages to create a sampling frame of eligible households: those with a household head, a caregiver, and a child under three years. Households were then randomly selected from this frame and invited to participate. Further details on the study design are available in the study protocol [34].

### Data collection

Data were collected approximately every 4–6 months across six survey waves using standardized structured questionnaires that were administered by trained research assistants to children’s primary caregivers or mothers [36–38]. The questionnaires, are available at the APHRC microdata portal, captured information on maternal and child nutrition (including anthropometry to assess wasting, stunting, and underweight); water, sanitation and hygiene conditions and practices; household shocks; child morbidity; and health-seeking behaviors. Study staff reviewed data daily to ensure the quality and completeness of the interviews.

The target sample size was 1544, but only 1211 households were enrolled at Wave 1. Sampling occurred over several months for each wave: Wave 1 (May–June 2021), Wave 2 (October–November 2021), Wave 3 (May–June 2022), Wave 4 (October–November 2022), Wave 5 (March 2023), and Wave 6 (August–September 2023). Community conflicts and household migrations contributed to participants’ loss to follow-up, which was accounted for in our sample size calculation [34].

### Dependent variable

The dependent variable of this study was maternal- or caregiver-reported child diarrhea (the passage of three or more loose or liquid stools in a day that is considered more frequent than normal) in the prior 2 weeks (yes/no) [39].

### Independent variables

**Fig 1:**
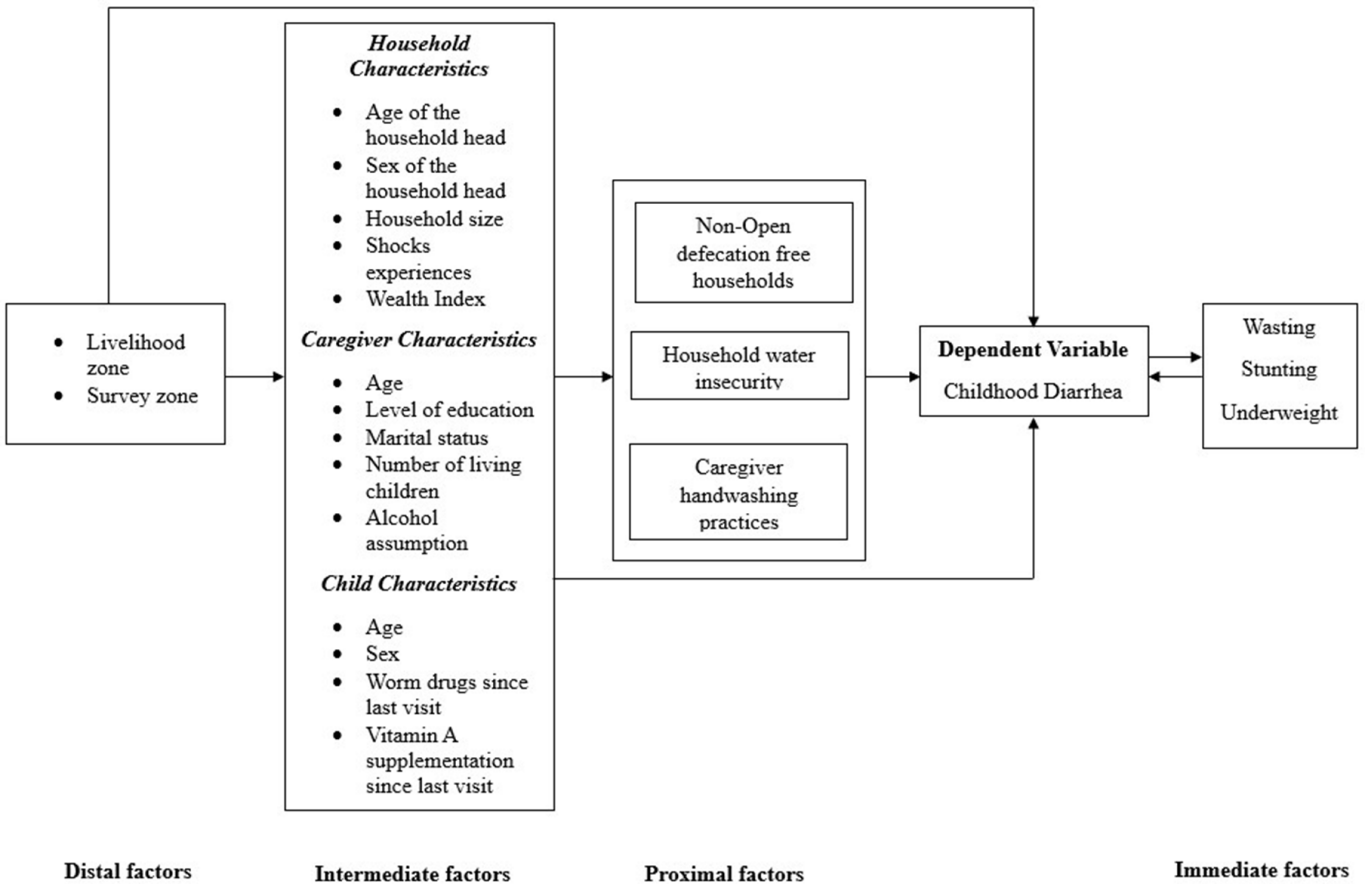
A conceptual framework for diarrhea risk factors among children under five.

Independent variables included distal, intermediate, and proximal factors, with wasting considered an additional independent variable given its bidirectional relationship with diarrhea (Fig 1). Distal factors included administrative and livelihood zones. Intermediate factors included head of household age (years), household head gender (man/woman), household wealth, number of household members, child age (months), child sex (male/female), child receipt of deworming drugs since the prior survey (yes/no), child receipt of vitamin A supplementation since the prior survey (yes/no), indicators of child nutritional status (wasting, stunting, and underweight; yes/no), caregiver age (years), caregiver education (formal/no formal education), caregiver marital status (in union: currently living together; not in union: separated, divorced, widowed or single), polygamy (yes/no), and reports household experience of climatic, biological, economic and conflict shocks in the four months before the survey. Caregivers self-reported current alcohol consumption (yes/no) was used as a proxy for inadequate caregiving practices, informed by baseline qualitative findings that identified alcohol as a contributor of suboptimal caregiving in Tukana. Proximal factors included water, sanitation, and hygiene (WASH) conditions and behaviors. Caregivers’ handwashing after visiting the toilet in the last 24 hours (yes/no) and household practice of open defection (yes/no) were based on self-report.

Livelihood zones were categorized as pastoral, agro-pastoral, fisherfolks, and urban/peri-urban. These were we further collapsed into three categories (pastoral, agro-pastoral, and urban/peri-urban/fisherfolk zones) given shared livelihood strategies across communities. Household wealth was determined based on a factor analysis of household assets, housing materials, and access to utilities, and divided into tertiles (low, medium, and high). Household shocks (yes/no) were categorized into four types: climatic (excessive rains/flooding and variable rain/drought), biological (livestock/crop/human disease outbreak, crop pest invasion, weed outbreak, and severe illness), economic (loss of livelihood, increased prices in food/agricultural/livestock inputs, loss of land/rental property, youth unemployment, death of a household member, delay in food assistance, delay in other safety net programs, and fire), and conflict (theft/destruction of assets, theft of livestock, domestic violence, and community conflicts).

Household water insecurity was measured using the 12-item Household Water Insecurity Experiences (HWISE) Scale [37]. Caregivers were asked to report how frequently in the prior 4 weeks they or others in their household experienced diverse water problems (0 = “never”, 1 = “rarely”, 2 = “sometimes”, 3 = “often” or “always”). Responses were summed, and households were categorized as experiencing no-to-marginal (scores of 0–2), low [3–11], moderate [12–23], or high [24–36] water insecurity [40]. Child nutrition indicators were assessed using wasting (weight-for-length/height z-scores (WHZ) <-2 SD), stunting (length/height-for-age z-score (HAZ) <-2 SD) and underweight (weight-for-age z-score (WAZ) <-2 SD) based on the 2006 World Health Organization child growth standards [41].

### Data analysis

Descriptive analyses were performed to examine the distribution of distal factors, household, caregiver, and child characteristics at Wave 1. We performed univariate analysis to assess the associations between the dependent variable (child diarrhea) and predictors of interest. Independent variables with a *p*-value <0.2 were retained and included in the final multivariable logistic regression models.

To identify risk factors of diarrhea, models were iteratively developed to account for the complex interrelationships between the different independent variables of interest, adjusting for either distal, intermediate, or proximal factors based on the conceptual framework in Fig. a1 [42]. Multivariable logistic regressions with generalized estimating equations and working correlation matrices were used to account for the within-subject correlation of repeated measurements across surveys [43,44]. Variables with *p*-value* *< 0.05 were associated with diarrhea. Adjusted odds ratios (AORs) and 95% confidence intervals (CIs) were obtained from the multivariable logistic regression models and interpreted for significant covariates.

All statistical analyses were performed using Stata 18 (Stata Corp, College Station, TX USA). The survey proportion command in Stata was used to compare each period and account for the complex sampling strategy. We performed a non-parametric Cochran-Armitage test for the trend in the prevalence across time using the nptrend command [45]. The xtgee command was used to perform univariate and multivariable logistic regressions.

### Ethical considerations

The study was approved by the African Medical and Research Foundation Ethical and Scientific Review Committee (Amref ESRC P905/2020) and the National Commission for Science, Technology, and Innovation of Kenya. The institutional review boards at the African Population and Health Research Center and Research Triangle Institute International signed a reliance agreement. Written informed consent was obtained from all caregivers of children under three years at baseline (Wave 1) and reaffirmed at each subsequent wave. Consent was provided through signature or thumbprint. Copies of the consent form were given to participants, retained by the study team, and securely stored in electronic format.

## Results

A total of 1211 eligible households were included in this study, and their baseline descriptive statistics are summarised in Table 1. Overall, 63.9% lived in the pastoral livelihood zone. About two-thirds (61.9%) of households were headed by men, while females headed more than a third (38.1%). At Wave 1, 77.3%, 92.8%, 90.6%, and 34.7% of households experienced biological, economic, climatic, and conflict shocks in the prior 4 months, respectively. Only 7.5% of the households were open-defecation free, and 44.3% were classified as having moderate water insecurity.

**Table 1:** Distribution of distal factors and household, caregiver, and child characteristics at baseline (Wave 1), Turkana County (n = 1,211).

Characteristic	Weighted (%)
**Distal factors**	
Livelihood zone	
Pastoral	63.9
Agro-pastoral	14.8
Urban/Peri-urban and Fisherfolk	6.5
Survey zone	
Central	21.0
North	25.6
South	24.1
West	29.2
**Household characteristics**	
Age of the household head (years)	
Less than 25	19.8
25-34	47.6
35 and above	32.6
Gender of household head	
Man	61.9
Woman	38.1
Household size^a^	6.1 ± 2.1
Wealth tertile	
Lowest	45.6
Middle	33.8
Highest	20.6
Experienced biological shock in the last 4 months	77.3
Experienced economic shock in the last 4 months	92.8
Experienced climatic shock in the last 4 months	90.6
Experienced conflict shock in the last 4 months	34.7
Household water insecurity	
No-to-marginal	11.8
Low	14.6
Moderate	44.3
High	29.2
Household open defecation free	7.5
**Caregiver characteristics**	
Age (years)	
Less than 25	10.8
25-34	34.5
35 and above	54.6
Education	
Formal education	13.6
No formal education	86.4
Marital status	
In union	85.2
Not in union	14.8
Polygamy	41.1
Number of living children	
Less than 3	25.9
3-5	49.6
6 or More	24.4
Current alcohol consumer	8.3
Practicing handwashing after using a toilet	78.4
**Child characteristics**	
Sex	
Male	54.8
Female	45.2
Age (months)^a^	17.3 ± 10.6
Wasting (weight for length/height z-score ≤ 2 SD)	
Yes	21.8
Stunting (length/Height for age z-score ≤ 2 SD)	
Yes	25.9
Underweight (weight for age z-score ≤ 2 SD)	
Yes	29.2
Child received deworming drugs in the prior 6 months	60.7
Child received vitamin A supplementation in the prior 6 months	45.8
Child experience diarrhea in the prior 2 weeks	32.1

^a^ Mean ± standard deviation.

Among caregivers, 47.6% were aged between 25–34 years, 86.4% had no formal education, 49.6% lived with 3–5 children, 85.2% were in a marital union, with 41.1% in polygamous marriage, 8.3% reported recently consuming alcohol, and 78.4% reported handwashing after using the toilet. Almost half of the children were boys (54.8%), with 21.8%, 25.9%, and 29.2% of children wasted, stunted, and underweight, respectively. Most (60.7%) of children received drugs for deworming in the prior 6 months and whereas only 45.8% received vitamin A supplementation in the same period. About one-third (32.1%) of caregivers reported that their child experienced diarrhea in the prior two weeks.

The overall prevalence of diarrhea and selected variables were examined at each period (Table 2). Child diarrheal prevalence decreased from 32.1% to 27.0% across the first year, followed by a further decrease at Wave 4. Between Waves 4 and 5, there was an increase from 15.7% to 17.8% in the prevalence of diarrhea. At the end of the study, the prevalence further decreased to 8.7%. A downtrend in prevalence of diarrhea was observed across livelihood zones, survey zones, and child sexes. A statistically significant downtrend in the prevalence of diarrhea was observed in the overall estimates, the livelihood zones, in the central and west survey zones, and among children younger than 36 months.

**Table 2:** Weighted prevalence of diarrhea by livelihood zone, survey zone, and child sex and age across survey waves (%, [95% CI]).

Variable	Wave 1 (N = 1211)	Wave 2 (N = 1068)	Wave 3 (N = 1019]	Wave 4 (N = 1134)	Wave 5 (N = 1082)	Wave 6 (N = 1042)	*p*-value^a^
Overall	32.1 [28.3, 36.1]	30.0 [26.0, 34.4]	27.9 [24.1, 32.1]	15.7 [12.5, 19.6]	17.8 [14.2, 21.9]	8.7 [6.3, 11.7]	<0.001
Livelihood zone							
Pastoral	32.6 [24.1, 36.3]	28.9 [21.9, 36.9]	20.7 [13.1, 31.1]	22.7 [16.9, 29.8]	18.5 [11.4, 28.6]	12.2 [7.8, 18.4]	< 0.001
Agro-pastoral	31.2 [26.4, 36.4]	27.8 [22.5, 33.7]	28.6 [24.0, 33.7]	12.5 [8.8, 17.5]	16.9 [12.7,22.2]	7.0 [4.3 11.3]	<0.001
Urban/Peri-urban and Fisher Folks	35.1 [28.5, 42.2]	39.5 [30.8, 48.9]	36.1 [28.1, 45.0]	21.5 [12.6, 34.5]	22.6 [17.3, 28.9]	11.3 [7.3, 17.2]	0.067
Survey zone							
Central	25.8 [20.4, 32.1]	32.8 [24.9, 41.7]	26.8 [20.3, 34.5]	18.4 [14.7, 22.9]	22.3 [18.0, 27.2]	9.4 [5.7, 14.9]	0.004
North	41.6 [33.1, 50.6]	22.5 [14.5, 33.2]	14.8 [8.7, 24.1]	22.1 [14.9, 31.5]	14.2 [7.6, 24.9]	14.1 [8.1, 23.5]	0.868
South	34.8 [27.5, 42.8]	37.9 [30.8, 45.5]	35.3 [28.6, 42.6]	17.3 [11.6, 24.9]	25.3 [18.2, 34.0]	8.2 [5.6, 11.9]	0.335
West	25.9 [20.4, 32.5]	28.2 [21.4, 36.2]	35.8 [30.6, 41.5]	6.9 [3.9, 12.3]	11.9 [7.2, 18.9]	2.9 [1.4, 6.1]	0.035
Child age (Months)							
0-23	31.4 [27.5, 35.7]	40.1 [33.4,47.1]	38.6 [33.1,44.5]	33.2 [25.8,41.5]	41.0 [31.2,51.6]	NA	0.015
24-35	31.6 [25.3,38.7]	30.5 [24.6,37.2]	27.3 [19.6, 36.6]	23.7 [17.2,31.7]	26.4 [20.9,32.9]	19.9 [13.4,28.5]	0.024
36-59	NA	20.4 [13.6,29.6]	20.9 [15.0,28.3]	21.5 [15.2,29.5]	15.9 [12.1, 20.5]	8.5 [6.2,11.6]	0.079
Child sex							
Male	33.9 [28.4, 40.0]	31.7 [26.1, 37.8]	26.2 [21.0, 32.2]	15.1 [11.1, 20.1]	18.9 [14.3, 24.4]	6.1 [4.0, 9.1]	0.294
Female	29.7 [24.5, 35.7]	28.2 [22.5, 34.7]	29.8 [24.1, 36.3]	16.5 [12.2, 21.9]	16.6 [12.2, 22.0]	11.5 [7.7, 16.9]	

^a^ Test for the trend in the prevalence of diarrhea across time.

Note: The study data collection period; Wave 1 (May/June 2021), Wave 2 (November 2021), Wave 3 (May/June 2022), Wave 4 (October/November 2022, Wave 5 (March 2023) and Wave 6 (August/September 2023).

In bivariate analyses, distal (livelihood and survey zones), intermediate (child age and sex, caregiver age, caregiver number of living children, caregiver alcohol consumption, head of household age, household shocks), proximal (household water insecurity, handwashing of caregiver after visiting toilet, household open defecation), and nutritional factors (wasting) were associated with diarrhea at *p* < 0.2 (S1 Table).

In the multivariable models (Table 3), children with caregivers who reported consuming alcohol had higher odds of diarrhea (adjusted odds ratio (AOR) = 1.28, 95% confidence interval (CI): 1.02-1.60) compared to those whose caregivers did not. Children in households that experienced any biological (AOR = 1.37, 95% CI: 1.20–1.57), climatic (AOR = 1.18, 95% CI: 0.93–1.50), or conflict shocks (AOR = 1.60, 95% CI: 1.40–1.83) also had higher odds of diarrhea compared to those in households that did not report experiencing such shocks in the prior 4 months. Relative to children living in households with no-to-marginal water insecurity, those in households with moderate (AOR = 1.25, 95% CI: 1.04–1.50) or high-water insecurity (AOR = 1.46, 95% CI: 1.19–1.81) had higher odds of diarrhea. Children in households that practiced open defecation had higher odds of diarrhea (AOR = 1.07, 95% CI: 0.91–1.26) compared to those in open defecation-free households. Children who were wasted (AOR = 1.20, 95% CI: 1.04–1.39), stunted (AOR = 1.40, 95% CI: 1.19–1.65), or underweight (AOR = 1.29, 95% CI: 1.11–1.49) also had significantly higher odds of diarrhea than those who were not.

**Table 3:** Factors associated with diarrhea among children under five in Turkana County, Kenya.

Variable	AOR [95% CI]	*p-value*
**Survey period** (ref: Wave 1)		
Wave 2	1.04 [0.89, 1.23]	0.605
Wave 3	0.90 [0.75, 1.07]	0.231
Wave 4	0.45 [0.37, 0.54]	<0.001
Wave 5	0.52 [0.43, 0.62]	<0.001
Wave 6	0.21 [0.17, 0.27]	<0.001
**Distal factors**		
**Livelihood zone (ref: urban/peri-urban and fisher folks)**		
Pastoral	0.90 [0.79, 1.03]	0.123
Agro-pastoral	1.10 [0.90. 1.34]	0.369
**Survey zone (ref: Central)**		
North	0.94 [0.74, 1.19]	0.602
South	1.17 [0.98, 1.41]	0.081
West	0.94 [0.78, 1.13]	0.519
**Intermediate factors (adjusted for distal factors)**		
**Child age (months)**	0.97 [0.96, 0.98]	< 0.001
**Child sex (ref: male)**		
Female	1.02 [0.88, 1.17]	0.833
**Worm drugs since last visit (ref: no)**		
Yes	0.88 [0.75, 1.02]	0.093
**Vitamin A supplementation since last visit (ref: no)**		
Yes	0.77 [0.66, 0.89]	0.001
**Caregiver age (years) (ref: below 25)**		
25-34	0.99 [0.81, 1.21]	0.959
35 and above	0.89 [0.70, 1.14]	0.345
**Caregiver number of living children (ref: < 3)**		
3-5	0.77 [0.64, 0.92]	0.005
6 or more	0.89 [0.70, 1.12]	0.318
**caregiver reported alcohol consumption (ref: no)**		
Yes	1.28 [1.02, 1.60]	0.028
**Household experienced any biological shocks in the prior 4 months (ref: no)**		
Yes	1.37 [1.20, 1.57]	<0.001
**Household experienced any climatic shocks in the prior 4 months (ref: no)**		
Yes	1.18 [0.93, 1.50]	0.171
**Household experienced any conflict shocks in the prior 4 months (ref: no)**		
Yes	1.60 [1.40, 1.83]	<0.001
**Proximal factors (adjusted for distal and intermediate factors)**		
**Household water insecurity (ref: no-to-marginal)**		
Low	1.19 [0.97, 1.47]	0.089
Moderate	1.25 [1.04, 1.50]	0.019
High	1.46 [1.19, 1.81]	<0.001
**Caregiver washes hands after using the toilet (ref: no)**		
Yes	0.83 [0.70, 0.98]	0.029
**Household open-defecation free (ref: yes)**		
No	1.07 [0.91, 1.26]	0.425
**Immediate factors (adjusted for distal, intermediate and proximal factors)**		
Wasted (WHZ < - 2 SD)	1.20 [1.04, 1.39]	0.013
Stunting (HAZ < -2 SD)	1.40 [1.19, 1.65]	<0.001
Underweight (WAZ < -2 SD)	1.29 [1.11, 1.49]	0.001

Note: The study data collection period: Wave 1 (May/June 2021), Wave 2 (November 2021), Wave 3 (May/June 2022), Wave 4 (October/November 2022, Wave 5 (March 2023) and Wave 6 (August/September 2023).

Children in households within the pastoral livelihood zone (AOR = 0.90, 95% CI; 0.79–1.03) had lower odds of diarrhea compared to those in the urban/peri-urban and fisher folk zones. Each one-month increase in child age was significantly associated with lower odds of diarrhea (AOR = 0.97, 95% CI: 0.96–0.98). Children who received deworming treatment had lower odds of diarrhea (AOR = 0.76, 95% CI: 0.66–0.86) than their counterparts who did not. Children whose caregivers were aged 35 years and above had lower odds of child diarrhea (AOR = 0.89, 95% CI: 0.70–1.14) compared with those aged 25 years and below. Households with a caregiver with 3–5 children had lower odds of diarrhea (AOR = 0.77, 95% CI: 0.64–0.92) than households with less than 3 children. Additionally, children whose caregivers reported washing their hands after using the toilet had significantly lower odds of diarrhea (AOR = 0.81, 95% CI: 0.69–0.96) than those whose caregivers did not.

## Discussion

In this study, we sought to assess the prevalence and risk factors of diarrhea among children in the drylands of Northern Kenya. In multivariable analyses, salient predictors of greater child diarrhea included caregiver alcohol consumption, household shocks, household water insecurity, open defecation, and child malnutrition. In contrast, livelihood zone, greater child age, expanded child deworming, increased child vitamin A supplementation, older caregiver age, lower number of children living in the household, and caregiver-reported handwashing after using the toilet were associated with lower odds of child diarrhea. These findings, together with previous studies, can help to inform policies and programs that aim to reduce the high incidence of child diarrhea in Kenya and similar drylands [46,47].

Households in the drylands of sub-Saharan Africa experience frequent shocks, protracted periods of poverty, and social and climatic vulnerabilities, which hinder access to food, water, and primary healthcare services. Studies in non-dryland settings have shown that shocks often exacerbate existing vulnerabilities, particularly among marginalized communities that rely on livestock for their livelihoods [48]. Families may struggle to maintain adequate food intake and access to clean water during such crises, resulting in child malnutrition, a key risk factor for child morbidity and mortality [49]. Our study complements this existing evidence by demonstrating that cumulative biological/health, climatic, and conflict shocks are associated with a higher risk of child diarrhea in arid and semi-arid areas [26,50].

Pastoral livelihood in sub-Saharan Africa increases vulnerability to shocks due to recurrent droughts and famines, leaving many dependents on food aid [51]. Some households adapt to this situation by diversifying livelihood strategies, including engaging in petty trade, crafting, weaving, and basketry. Research has shown that when pastoral households lose livestock, children are particularly vulnerable to illnesses such as diarrhea and respiratory infections, primarily due to poor nutrition and limited access to primary healthcare services [52]. Our recent study indicates that pastoral livelihood might be a protective factor against childhood diarrhea. This finding contradicts previous research and opens a new question for future investigation.

Our study revealed that the prevalence of diarrhea declined across survey waves, which may partly be attributed to the aging of the cohort over time, as older children typically have more developed immune systems and enhanced resistance to enteric infections. Greater caregiver age and larger household size were also associated with lower odds of diarrhea, potentially reflecting increased caregiving experience and improved hygiene awareness among older caregivers and within larger households. Studies have reported an association between greater vitamin A supplementation and reduced child diarrhea incidence [53]. Deworming of children with anti-parasitic medication and adoption of periodic deworming campaigns has been strongly recommended to prevent diarrhea [54]. This study similarly suggests that these efforts may be protective against child diarrhea.

Caregivers who reported alcohol consumption had significantly higher odds of experiencing diarrhea. This aligns with prior studies that have found caregiver alcoholism to be associated with persistent malnutrition in children under five [55]. Consumption of alcohol by caregivers with children may affect caregiving practices, particularly those related to hand hygiene during the preparation of food and child feeding [56]. This is an open area of research on the effect of alcohol consumption on caregiving practices in the drylands of sub-Saharan Africa.

Greater experiences of household water insecurity (which includes issues with water availability, accessibility, and sufficiency for diverse household uses) have been similarly associated with greater diarrhea risk among children under five in Ethiopia [57] and Nigeria [58]. Most studies, however, have only examined child diarrhea related to directly observable, supply-side water indicators, such as drinking water source and quality [59,60]. Our findings demonstrate that experiential measures of water insecurity provide complementary information to traditional water metrics and make clear that water insecurity is an important modifiable risk factor that should be targeted to improve child health and well-being.

As hypothesized, we found that undernutrition was significantly associated with higher odds of diarrhea infections in children. Malnutrition undermines child development by depleting nutritional reserves and compromising the immune system, thereby increasing susceptibility to diarrheal infections [61]. Diarrhea, in turn, can exacerbate malnutrition, leading to a bidirectional relationship between these two conditions that ultimately increases the risk of child mortality [62].

Our study had several limitations. First, caregiver-reported child diarrhea is subject to recall bias. Further, we did not ask about the type of diarrhea observed (e.g., acute watery, acute bloody, or persistent), each of which might have different health impacts. Systematic reviews have, however, noted a lack of evidence supporting the validity of instruments used to classify diarrhea in pediatric populations [63]. As such, future research is needed to develop and validate better child diarrhea measures so that analyses can be disaggregated by diarrhea subtype. Second, some households in these settings are nomadic or semi-nomadic, and highly mobile families may have been underrepresented if they were not present in selected villages at the time of enumeration, potentially introducing selection bias. Third, this longitudinal study was not designed to assess diarrhea as a primary outcome, although the large sample size reduces the risk of underpowered analyses. Lastly, the surveys were conducted during an extended dry season, with many households experiencing climatic and economic shocks, which contributed to the high prevalence of diarrhea and wasting in children.

## Conclusion

Child age, child wasting, caregiver age, caregiver alcohol use, household shocks, and household water insecurity were associated with diarrhea risk among young children in Turkana, Kenya. Our findings suggest that a multisectoral approach may be effective at reducing the burden of child diarrhea in this region. Such an approach should prioritize interventions that expand access to improved water, sanitation, and hygiene services; prevent and manage child malnutrition; reduce alcohol consumption among caregivers; and improve household resilience to manage biological/health, climatic, and conflict shocks. Lastly, community-wide deworming and vitamin A supplementation campaigns could reduce the incidence of child diarrhea and improve both child growth and well-being. Future studies should consider clinical assessments to confirm cases of child diarrhea or design ways to validate self-reported practices with observational data.

## Supporting information

S1 Table Bivariate logistic regression analysis of factors associated with diarrhea among children under five years of age. (DOCX)
