# Supplementary material for: Prevalence and risk factors of diarrhea among young children in Kenya’s drylands: A longitudinal study
Source: PLOS Glob Public Health. 2026 Jan 8;6(1):e0003998. doi: 10.1371/journal.pgph.0003998 (PMC12782384; doi:10.1371/journal.pgph.0003998)
Supplement: S1 Table — (DOCX) [file pgph.0003998.s001.docx]

**S1 Table.** **Factors associated with diarrhea among children under five in univariate logistic regressions.**

| Variable | Crude OR [95% CI] | P-value |  |
| --- | --- | --- | --- |
| **Distal factors** |  |  | |
| Livelihood zone (Ref. non-pastoral) |  |  | |
| Pastoral | 0.72 [0.63, 0.81] | <0.001 | |
| Administrative zone (Ref: Central) |  |  | |
| North | 0.93 [0.74, 1.17] | 0.549 | |
| South | 1.16 [0.97, 1.37] | 0.087 | |
| West | 0.91 [0.77, 1.09] | 0.304 | |
| **Intermediate factors** |  |  | |
| ***Child characteristics*** |  |  | |
| Age (months) | 0.96 [0.95, 0.96] | <0.001 | |
| Female (Ref: male) | 1.03 [0.91, 1.18] | 0.617 | |
| ***Caregiver characteristics*** |  |  | |
| Age (years) | 0.98 [0.97, 0.98] | <0.001 | |
| Formal education (Ref: non-formal education) | 0.85 [0.73, 1.00] | 0.051 | |
| Not in a marital union (Ref: in union) | 1.00 [0.85, 1.18] | 0.955 | |
| Number of living children (Ref: < 3) |  |  | |
| 3-5 | 0.72 [0.62, 0.84] | <0.001 | |
| 6 or more | 0.75 [0.62, 0.90] | 0.002 | |
| Report alcohol consumption (Ref: no) | 1.32 [1.07, 1.62] | 0.009 | |
| ***Household characteristics*** |  |  | |
| Age of the household head | 0.99 [0.99, 1.00] | 0.001 | |
| Female head of household (Ref: Male) | 0.96 [0.84, 1.09] | 0.529 | |
| Household size | 1.01 [0.98, 1.04] | 0.634 | |
| Owns a radio or television (Ref: non) | 0.97 [0.79, 1.19] | 0.755 | |
| Wealth tertile (Ref: lowest) |  |  | |
| Middle | 1.08 [0.92, 1.26] | 0.362 | |
| Highest | 1.09 [0.93, 1.28] | 0.303 | |
| Shocks experienced in the last 4 months before the survey (Ref: less than two) |  |  | |
| Two | 1.14 [0.84,1.55] | 0.408 | |
| Three | 1.84 [1.36, 2.48] | <0.001 | |
| Four | 2.56 [1.88, 3.47] | <0.001 | |
| **Proximal factors** |  |  | |
| Household water insecurity (Ref: no-to-marginal) |  |  | |
| Low | 1.25 [1.03, 1.52] | 0.027 | |
| Moderate | 1.32 [1.11, 1.56] | 0.002 | |
| High | 1.48 [1.23, 1.78] | <0.001 | |
| Caregiver wash hands after using the toilet (Ref: no) |  |  | |
| Yes | 0.89 [0.76, 1.04] | 0.144 | |
| Household practised open defecation (Ref: No) |  |  | |
| Yes | 1.22 [1.04, 1.42] | 0.012 | |
| **Immediate factor** |  |  | |
| Child Wasting (ref: no) | 1.22 [1.06, 1.40] | 0.006 | |
